# Durable response to EGFR tyrosine kinase inhibitors in a patient with non–small cell lung cancer harboring an EGFR kinase domain duplication

**DOI:** 10.1111/1759-7714.14081

**Published:** 2021-07-09

**Authors:** Esuteru Hirokawa, Satomi Watanabe, Kazuko Sakai, Masayuki Takeda, Chihiro Sato, Takayuki Takahama, Kazuto Nishio, Kazuhiko Nakagawa

**Affiliations:** ^1^ Department of Medical Oncology Kindai University Faculty of Medicine Osaka Japan; ^2^ Department of Genome Biology Kindai University Faculty of Medicine Osaka Japan

**Keywords:** carcinomatous meningitis, epidermal growth factor receptor (EGFR), kinase domain duplication, tyrosine kinase inhibitor (TKI)

## Abstract

Epidermal growth factor receptor (EGFR) kinase domain duplication (KDD) has been identified as an oncogenic driver in 0.05% to 0.14% of non–small cell lung cancer (NSCLC) patients. However, little is known of the efficacy of EGFR tyrosine kinase inhibitors (TKIs) for such patients. Here, we report the case of a 45‐year‐old Japanese woman with NSCLC positive for EGFR‐KDD (duplication of exons 18–25) who developed carcinomatous meningitis and showed a marked response to the EGFR‐TKIs erlotinib and osimertinib. As far as we are aware, this is the first report of EGFR‐TKI efficacy for carcinomatous meningitis in a NSCLC patient harboring EGFR‐KDD.

## Graphical abstract

We report a first case of a patient with NSCLC positive for EGFR kinase domain duplication (duplication of exons 18–25) who developed carcinomatous meningitis and showed a marked response to the EGFR‐TKIs erlotinib and osimertinib.

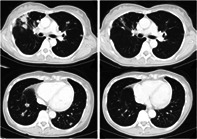

## INTRODUCTION

Tyrosine kinase inhibitors (TKIs) are established as standard therapy for non–small cell lung cancer (NSCLC) with sensitizing mutations of the epidermal growth factor receptor (EGFR).^1^, ^2^ EGFR kinase domain duplication (EGFR‐KDD) is the result of rare genomic alterations that activate EGFR signaling and confer sensitivity to EGFR‐TKIs.^3^, ^4^, ^5^, ^6^, ^7^, ^8^, ^9^, ^10^, ^11^ Most instances of EGFR‐KDD are the result of duplication of exons 18 to 25 of *EGFR*, although rare cases due to duplication of exons 17 to 25 or exons 14 to 26 have been described.^4^ Such genomic alterations in NSCLC are thought to occur at a frequency of 0.05% to 0.14%.^11^ Here, we report a rare case of a patient with NSCLC harboring duplication of exons 18 to 25 of *EGFR* who experienced benefit from treatment with the EGFR‐TKIs erlotinib and osimertinib. Carcinomatous meningitis of the patient showed marked resolution during osimertinib treatment, which represents the first such successful treatment to be reported in an individual with EGFR‐KDD.

## CASE REPORT

**FIGURE 1:**
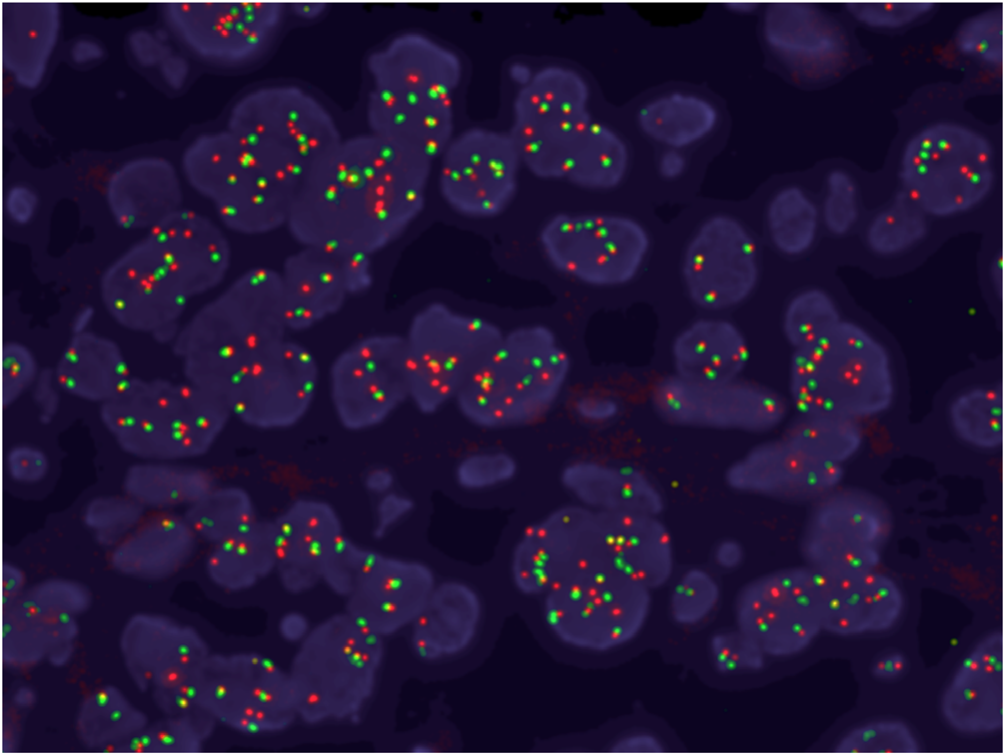
Fluorescence in situ hybridization (FISH) analysis showed amplification of *EGFR* (copy number of 5.85) in a lymph node biopsy specimen obtained before EGFR‐TKI treatment. Red and green signals indicate *EGFR* gene and centromere 7 (CEP7) respectively

**FIGURE 2:**
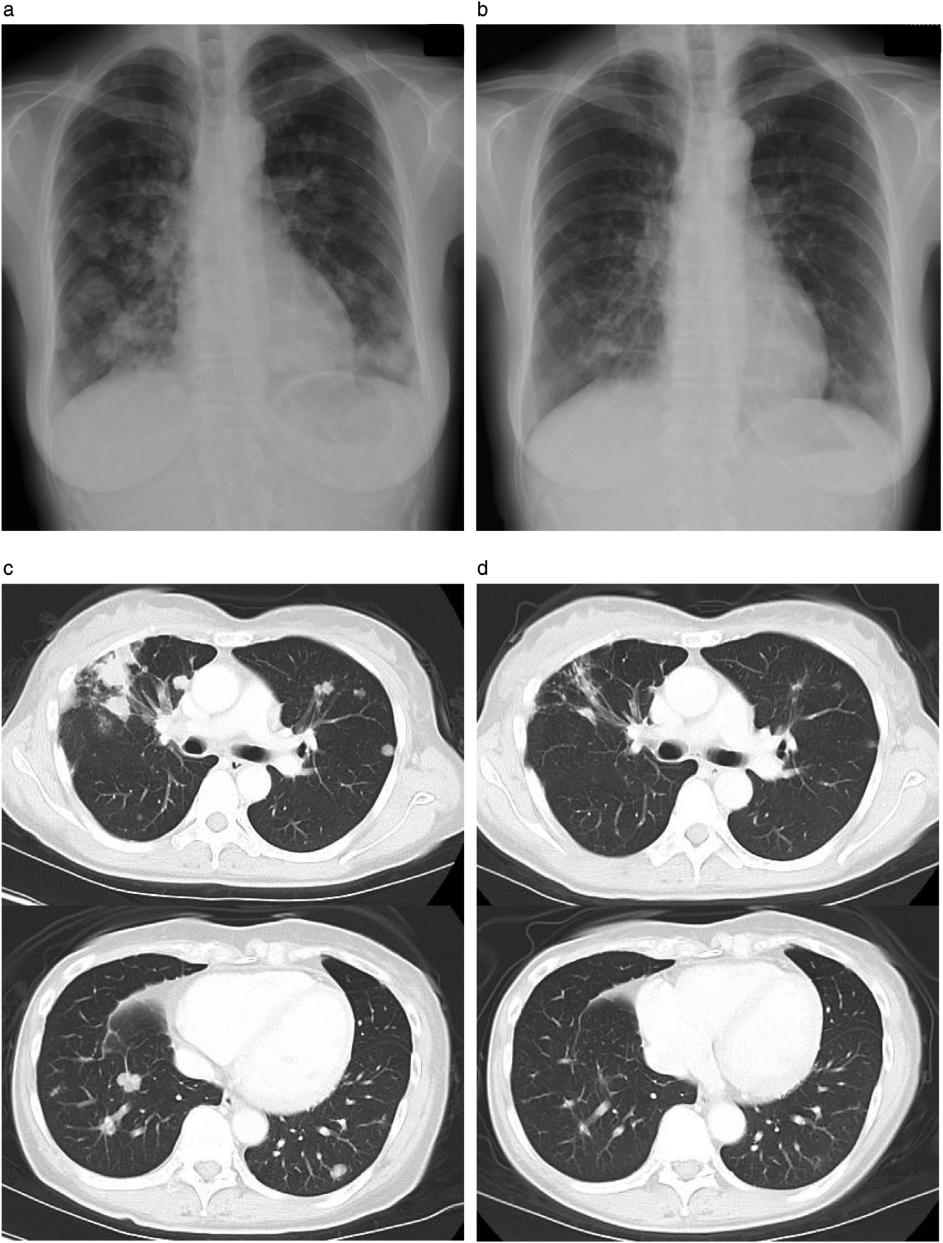
Chest x‐rays revealed a marked reduction in the size of lung metastases at two weeks after initiation of erlotinib treatment (a, b), whereas chest CT scans at 70 days showed a partial response with a reduction in the size of multiple lung metastases (c, d)

A 45‐year‐old Japanese woman without a history of smoking was referred to our hospital for the treatment of recurrent NSCLC with multiple lung and mediastinal lymph node metastases. A supraclavicular lymph node biopsy revealed adenocarcinoma. Routine screening for *EGFR* mutations (cobas EGFR mutation test v2, Roche Molecular Diagnostics), *ALK* fusion genes, and *ROS1* fusion genes was negative. The tumor proportion score for programmed cell death–ligand 1 (PD‐L1, 22C3) was 20%. Next‐generation sequencing (NGS) with an Ion AmpliSeq Custom DNA Panel (Genomedia) identified *EGFR* amplification (copy number of 5.85), which was confirmed by fluorescence in situ hybridization (FISH) (Figure 1). To further investigate the gene alteration, we performed *EGFR* sequencing using the Sanger method, and duplication of introns 17 to 25 was detected. After the patient had received carboplatin‐pemetrexed combination therapy, pembrolizumab monotherapy, gamma knife stereotactic radiosurgery for asymptomatic multiple brain metastases, and docetaxel‐ramucirumab combination therapy, she was started on erlotinib (150 mg/day) in the fourth‐line setting. Fourteen days after the onset of erlotinib treatment, chest x‐rays revealed a pronounced reduction in the size of the lung metastases (Figure 2(a), (b)). At 70 days after treatment onset, computed tomography (CT) also showed a reduction in the size of multiple lung metastases (Figure 2(c), (d)), which was categorized as a partial response (PR) according to Response Evaluation Criteria in Solid Tumors (RECIST) version 1.1. The patient experienced progressive disease for lung metastases at 133 days after erlotinib initiation, although no progression was apparent for brain metastases during the treatment period.

**FIGURE 3:**
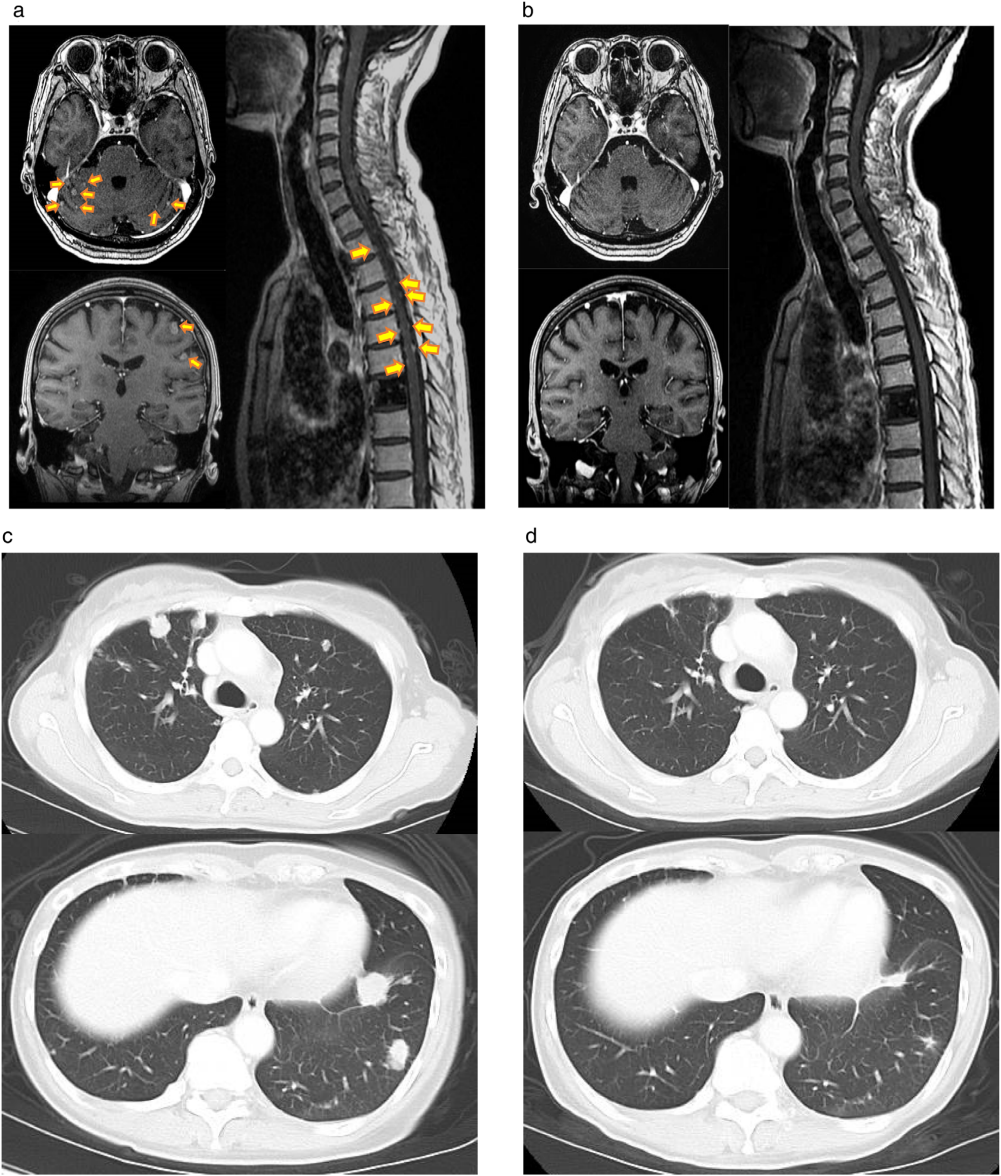
Brain and spinal cord MRIs (contrast‐enhanced, T1‐weighted imaging) revealed carcinomatous meningitis (arrows) before osimertinib therapy (a) and pronounced amelioration of this condition at 29 days after therapy onset (b), whereas CT showed a reduction in the size of pulmonary lesions at 55 days after the onset of osimertinib treatment (d) compared with baseline (c)

CT‐guided percutaneous lung puncture was performed, and NGS with an Ion AmpliSeq Cancer Hotspot Panel v2 (ThermoFisher Scientific) found no gene alterations of note, while *EGFR* amplification (copy number of 4.3) remained evident by FISH. After she had experienced progression on an investigational therapy and S‐1 monotherapy, the patient complained of numbness in her extremities. Contrast‐enhanced, T1‐weighted magnetic resonance imaging (MRI) of the head revealed diffuse leptomeningeal contrast enhancement following the contours of the gyri and sulci of cerebellar folia and multiple deposits in the subarachnoid space, whereas MRI of the spine revealed nodular enhancement along the cord surface (Figure 3(a)). The patient was therefore diagnosed with carcinomatous meningitis and was started on osimertinib (80 mg/day) in the seventh‐line setting. Her weight and body surface area on day 1 were 46 kg and 1.423 m^2^, respectively. Her Eastern Cooperative Oncology Group performance status (PS) was 2, and physical examination showed no abnormalities. Laboratory data were almost normal, other than grade 1 hyponatremia (Common Terminology Criteria for Adverse Events [CTCAE] version 4.0). Her symptoms of numbness was rapidly relieved, and her PS had improved to 0 by day 14. She achieved a PR at extracranial sites (Figure 3(c), (d)). MRI on day 29 revealed a complete response of the central nervous system metastases (Figure 3(a), (b)). Rebiopsy of lung metastatic tissue was performed during osimertinib treatment, and genomic profiling of the specimen with the FoundationOne CDx Panel (Foundation Medicine) showed the EGFR‐KDD of introns 17 to 25, as well as *TP53* R65fs*58, *FGFR4* R248Q, *ATRX* R498G, and *VEGFA* S186F mutations. Osimertinib was effective overall for 14.5 months, after which follow‐up MRI of the head showed progression of brain metastases. Treatment was changed to afatinib, and the patient was transferred to another hospital, where she continued afatinib therapy for one month but experienced clinical progression with deterioration of performance status. She then received best supportive care and died two months after afatinib initiation. Her overall survival was 44 months from the start of first‐line therapy with the carboplatin‐pemetrexed regime.

## DISCUSSION

Here, we describe the case of a patient with EGFR‐KDD who responded to erlotinib and subsequently to osimertinib. NGS of a tumor specimen obtained after erlotinib therapy did not reveal a potential resistance mechanism, including the *EGFR* T790M mutation. The limited data available for the treatment of NSCLC patients with EGFR‐KDD has shown that it is sensitive to several EGFR‐TKIs.^3^, ^4^, ^5^, ^6^, ^7^, ^8^, ^9^, ^10^, ^11^ Preclinical studies showed that afatinib inhibited the growth of cells expressing an EGFR‐KDD form of the receptor and that cetuximab further enhanced the activity of afatinib.^6^, ^11^ An in silico study suggested that osimertinib occupies the ATP binding site of EGFR‐KDD more stably compared with gefitinib and afatinib.^12^ In the case reported here, osimertinib was effective after the development of resistance to erlotinib, possibly reflecting the high binding affinity of osimertinib for the kinase domain of EGFR.

Osimertinib achieved marked disease control for carcinomatous meningitis of the proband. A recent report described the efficacy of osimertinib in an EGFR‐KDD–positive NSCLC patient with brain metastasis.^10^ However, as far as we are aware, no study has previously reported the efficacy of osimertinib for carcinomatous meningitis in a patient with EGFR‐KDD.

In the current case, EGFR‐KDD was detected by Sanger sequencing and the FoundationOne CDx Panel but not by polymerase chain reaction (PCR) analysis or NGS with the Ion AmpliSeq Cancer Hotspot Panel v2. Given that most conventional sequencing platforms or PCR‐based methods do not routinely detect *EGFR* alterations affecting introns, such screening procedures may miss some patients who would benefit from targeted therapy. We reviewed 19 EGFR‐KDD patients from seven studies and the case reported here (*n* = 20 in total) in order to investigate who should further be examined for EGFR‐KDD.^3^, ^4^, ^5^, ^6^, ^7^, ^9^, ^10^ Patient ages ranged from 33–87 (median, 59.5), and 12/21 (57.1%) patients were male. In cases with smoking history status, all the patients were non‐ or light‐smokers; five out of six (83.3%) were non‐smokers, and one out of six patients (16.7%) was a light‐smoker (one pack‐year). Five out of 14 patients (35.7%) with EGFR‐KDD simultaneously harbored *EGFR* amplification.^4^ Given those patient characteristics, non‐ or light‐smokers without any known driver gene alteration might be candidates for further examination targeting EGFR‐KDD. *EGFR* amplification without known *EGFR* mutation might also suggest the existence of EGFR‐KDD. In such cases, using a NGS panel designed to detect *EGFR* rearrangement occurring in intron such as FoundationOne CDx or MSK IMPACT (Memorial Sloan Kettering Cancer Center) should be considered.

In conclusion, we report a case of NSCLC, positive for EGFR‐KDD, that showed a notable response to two EGFR‐TKIs. A complete response of carcinomatous meningitis was achieved with osimertinib treatment. Further studies are warranted to clarify the best treatment strategy for patients with EGFR‐KDD.

## CONFLICT OF INTEREST

Takayuki Takahama, Kazuko Sakai, Masayuki Takeda, Kazuto Nishio, and Kazuhiko Nakagawa received honoraria from AstraZeneca K.K. and Chugai Pharmaceutical Co., Ltd. Kazuhiko Nakagawa discloses financial research support from AstraZeneca K.K. and Chugai Pharmaceutical Co., Ltd. The other authors declare no conflicts of interest. There was no support in the form of grants, gifts, equipment, or drugs for this study.
